# Association of C-Reactive Protein and Metabolic Disorder in a Chinese Population

**DOI:** 10.3390/ijerph120708228

**Published:** 2015-07-17

**Authors:** Mingxia Sun, Liying Zhang, Shanying Chen, Xinyu Liu, Xiaofei Shao, Hequn Zou

**Affiliations:** 1 Department of Nephrology, The Third Affiliated Hospital of Southern Medical University, Guangzhou, Guangdong 510630, China; E-Mails: feixia_97@163.com (M.S.); zhangliying926@126.com (L.Z.); dqydqy386@sina.com (S.C.); pepsi84@163.com (X.L.); shaoxfei@126.com (X.S.); 2 Department of Nephrology, Huhhot First Hospital, Huhhot, Inner Mongolia 010010, China; 3 Department of Nephrology, The First Affiliated Hospital of Inner Mongolia Medical University, Huhhot, Inner Mongolia 010010, China; 4 Department of Nephrology, Zhangzhou Affiliated Hospital of Fujian Medical University, Zhangzhou, Fujian 363000, China

**Keywords:** C-reactive protein, metabolic disorder

## Abstract

*Objective*: To assess the high-sensitivity C-reactive protein (hs-CRP) levels and explore the risk factors for an elevated hs-CRP level. We also provide the clinical utility of CRP to identify subjects with metabolic syndrome (MetS). *Methods*: Data were drawn from a cross-sectional survey in China. Subjects were divided into three subgroups: hs-CRP ≤ 1 mg/L, 1 mg/L < hs-CRP ≤ 3 mg/L and hs-CRP > 3 mg/L. Multiple linear regressions and logistic regression models were used. *Results*: In the Chinese population, 50.43% subjects had a low hs-CRP level, 30.21% subjects had an intermediate hs-CRP level and 19.36% subjects had an elevated hs-CRP level. Age, physical inactivity, abdominal obesity, a low LDL level, an elevated fasting glucose level, uric acid and urinary albumin to creatinine ratio (ACR) were correlated with log-CRP. In multivariate analysis, relative risks of an elevated CRP level were 2.40 (95% CI 1.44–3.99, *p* = 0.001), 3.63 (95% CI 2.20–5.98, *p* < 0.001), 4.23 (95% CI 2.51–7.11, *p* < 0.001) and 6.23 (95% CI 3.45–11.26, *p* < 0.001) for subjects with 1, 2, 3, or more than 3 MetS components, respectively. The accurate estimates of the area under the receiver operating characteristic of hs-CRP for MetS was 0.6954 (95% CI, 0.67–0.72). *Conclusion*: Age, physical inactivity, abdominal obesity, a low LDL level, an elevated fasting glucose level, uric acid and ACR are correlated with log-CRP. The number of MetS components is a significant determinant of elevated CRP levels after adjusted for other potential confounders.

## 1. Introduction

According to the findings from the Global Burden of Disease Study 2010, cardiovascular disease (CVD) is the leading cause of death in China [1]. Hypertension, diabetes, dyslipidemia, older age, male sex and smoking are well known CVD risk factors [2,3]. Evaluation of CVD risk factors and early intervention might contribute to lower mortality from CVD. In addition, C-reactive protein (CRP), one acute-phase protein correlated with inflammation, has been considered as a novel risk factor of CVD [2]. Several prospective studies have demonstrated that high-sensitivity CRP (hs-CRP) independently predicts vascular events and adds predictive value to the Framingham Risk Score [2]. It is also found that hs-CRP levels are correlated with metabolic syndrome (MetS) [4,5,6,7,8,9,10,11], and endothelial dysfunction [12]. 

Based on this evidence, the CDC-AHA Writing Group recommended the optional use of hs-CRP testing in patients with 10-year Framingham coronary heart disease (CHD) risk between 10% and 20% [13]. It is recommended that a hs-CRP level of less than 1.0 mg/L is considered to denote low risk, 1.0 to 3.0 mg/L intermediate risk, and greater than 3.0 mg/L high risk [14,15]. CRP is also proposed as one component of MetS [4,16]. However, previous studies are based on western countries. One previous study [3] based on a Japanese population suggested that a tentative cut point of hs-CRP as a component of MetS might be 0.45 mg/L in men and 0.25 mg/L in women. The results indicated the ethnic differences in the CRP levels [3]. There may also be ethnic differences in the clinical utility of CRP. As we know, there are relatively limited data on CRP in Chinese individuals. In our previous study, the result indicated that obesity is associated with the level of CRP, but the associations of other metabolic disorders and the level of CRP were not reported in the study [16]. In this previous study, we have assessed an elevated level of CRP in the obese and non-obese subgroups, however, the data of a low level and an intermediate level of CRP were not applied [16]. We carried the present study to assess the CRP levels in a Chinese population and explore the risk factors of an elevated CRP level. We also provide the clinical utility of CRP to identify men and women with MetS.

## 2. Experimental Section

### 2.1. Ethics Statement

This study was approved by the ethics committee of the Third Affiliated Hospital of Southern Medical University. All subjects gave their written informed consent. This study was supported by the following Science Foundation: (1) EU FP7 Program, UroSense, 2011; (2) ISN Research Committee grant, 2007; (3) ISN Research Committee grant, 2004; (4) Guangdong Provincial Science and Technique Program (No. 2011B031800386), 2011. 

### 2.2. Study Population

We used the database drawn from one cross-sectional survey which was conducted in Wanzhai Town, Zhuhai City, China. This survey was performed from June 2012 to October 2012. Finally, 2142 residents (aged 18 years or older) completed the survey. A total of 308 subjects were excluded because of missing data. A total of 1843 subjects were included in the present analyses. We have described the survey in previous article [17]. 

### 2.3. Data Collection

Data on socio-demographic status, personal and family health history and lifestyle habits were obtained through questionnaires. History of drug use was also obtained by questionnaires. Systolic blood pressure (SBP) and diastolic blood pressure (DBP) were determined on the right arm in a seated position using a calibrated mercury sphygmomanometer. Before blood pressures were determined, all of the subjects rested for at least 5 min. Blood pressures were determined consecutively three times and the average of the three readings was calculated [17]. Waist circumference was measured according to the World Health Organization recommended protocols [18]. 

### 2.4. Determination of CRP

High sensitivity C-reactive protein (hs-CRP) was determined using an enzymatic immunoassay turbidimetric method (reagent: Orion, Finnish; apparatus: Roche Cobas 6000, Penzberg, Germany). After an overnight fasting for at least 10 h, blood specimens were collected. All blood specimens were analyzed in the central laboratory of Third Affiliated Hospital of Southern Medical University [17]. According to the previous guideline, a hs-CRP level of less than 1.0 mg/L is considered to a low level, 1.0 to 3.0 mg/L an intermediate level, and greater than 3.0 mg/L an elevated high risk [14,15].

### 2.5. Metabolic Disorder

According to Third Report of the National Cholesterol Education Program (NCEP) Expert Panel, MetS was defined as having at least three of the five components: abdominal obesity, elevated triglyceride levels (≥150 mg/dL), low high density lipoprotein cholesterol levels (<40 mg/dL in men, or <50 mg/dL in women), an elevated blood pressure (≥130/85 mmHg) and an elevated fasting glucose level (≥6.1 mmol/L) [19]. Abdominal obesity was modified for Chinese as >90 cm for men and >80 cm for women based on the definition of MetS by International Diabetes Federal [20]. Hyperuricemia was defined as serum uric acid ≥ 7 mg/dL in men or ≥ 6 mg/dL in women [21,22]. Albuminuria is also an independent predictor of CVD in both men and women [23]. Albuminuria was defined as urinary albumin to creatinine ratio (ACR, mg/g) ≥ 30 mg/g [24]. Urinary albumin to creatinine ratio (ACR, mg/g) was calculated as the ratio of urinary albumin to urinary creatitine. Serum creatinine, serum fasting glucose, serum total cholesterol, serum triglyceride, serum high-density lipoprotein cholesterol (HDL), serum low-density lipoprotein cholesterol (LDL) and urinary creatitine were determined using colorimetric methods. Serum insulin was determined using electrochemiluminescence immunoassays. Urinary albumin was measured by an immune nephelometric method [17]. 

Homeostatic model assessment of insulin resistance (HOMA-IR) was calculated as fasting plasma glucose (mmlo/L) × fasting insulin (mU/L)/22.5 [25]. Insulin resistance was defined as HOMA-IR > 2.69 (exceeding the 75% percentile of HOMA-IR in normal glucose tolerance subjects) [26].

### 2.6. Data Analysis

Using Stata (version 11) for data analysis, 1834 subjects were included in the analysis. Continuous variables were reported as mean and standard deviation or median values and interquartile range as appropriate. Categorical data were presented by frequencies and percentages. Based on the levels of hs-CRP, participants were divided into three subgroups for all analyses: participants with a low level of hs-CRP (hs-CRP ≤ 1 mg/L); participants with an intermediate CRP level (hs-CRP > 1 mg/L and hs-CRP ≤ 3 mg/L); participants with en elevated CRP level (hs-CRP > 3 mg/L). One-way Analysis of Variance, or the Kruskal-Wallis test, was used to analyze the differences in clinical characteristics for three subgroups if the variables were continuous variables. A Bonferroni’s adjustment was used for the effect of the multiple comparisons. The chi-squared test or Fisher’s exact test was used to compare categorical variables.

In order to examine the risk factors associated with hs-CRP, multiple linear regressions were used. High sensitivity C-reactive protein was used as an independent variable. All variables with skewed distributions were logarithmically transformed before being analyzed. These variables included hs-CRP, serum triglyceride and ACR. Firstly, variables were examined in unadjusted models. These variables included: socio-demographic characteristics (age, sex, and education attainment), comorbidities (history of coronary heart disease, and history of stroke), lifestyle factors (current smoker, current alcohol use, and physical inactivity), components of MetS, serum uric acid and ACR. Next all of these variables were examined in the adjusted model. Only eight subjects were taking statin, so taking statin was not added into the adjusted model. 

We also examined the associations of the level of CRP with the number of MetS components present (0, 1, 2, 3, and more than 3). An elevated CRP level was defined as the hs-CRP level > 3 mg/L. Logistic regression models were used and adjusted for the covariates used in the above regression models. Subjects with 0 component of MetS were designated as the reference subgroup. 

Accurate estimates of the area under the receiver operating characteristic (AUROC) curve analysis was performed by using the level of CRP for diagnosing MetS. An AUROC ≥ 0.7 was considered as acceptable [27]. Youden’s index was used to select the optimal cut-offs for the level of CRP. Youden’s index was calculated as (specificity + sensitivity − 1).

## 3. Results and Discussion

### 3.1. Baseline Characteristics (Table 1)

Baseline characteristics of the subjects are presented in the Table 1. 1834 subjects (the mean age was 52.8 ± 14.5 years and 679 were men) were included in the analysis. The median hs-CRP was 0.99 mg/L (interquartile range, 0.46–2.37 mg/L) in the total population. The median hs-CRP was 1.01 mg/L (interquartile range, 0.49–2.63 mg/L) in men and 0.98 mg/L (interquartile range, 0.44–2.25 mg/L) in women, respectively [28]. There was no significantly difference in CRP between men and women (*p* = 0.17, not shown in the table) [28].

**Table 1:** Characteristics of subjects according to the level of C-reactive protein.

Clinical Characteristics	Group 1	Group 2	Group 3	*p* Value
	CRP ≤ 1 mg/L	1 mg/L < CRP ≤ 3 mg/L	CRP > 3 mg/L	
	N = 925	N = 554	N = 355	
C-reactive protein	0.47 (0.28–0.69)	1.69 (1.29–2.19)	5.18 (3.73–8.31)	<0.001
Age (Years)	50.02 ± 14.71	54.87 ± 13.65	56.67 ± 13.87	<0.001
Male (%)	338 (36.5)	198 (35.9)	142 (40.1)	0.42
History of diabetes mellitus (%)	44 (4.8)	38 (6.9)	33 (9.3)	<0.001
History of hypertension (%)	127 (13.8)	145 (26.3)	100 (28.1)	<0.001
History of stroke (%)	3 (0.3)	2 (0.4)	2 (0.6)	0.78
History of coronary heart disease (%)	19 (2.1)	10 (1.8)	13 (3.7)	0.16
Current smoker (%)	93 (10.3)	76 (13.8)	54 (15.3)	0.02
Current alcohol use (%)	45 (4.9)	38 (6.9)	23 (6.4)	0.23
High school or above (%)	448 (48.4)	197 (35.7)	116 (32.46)	<0.001
Physical inactivity (%)	502 (54.27)	320 (57.8)	191 (53.8)	0.36
Systolic blood pressure (mmHg)	124.16 ± 18.47	131.95 ± 20.82	134.93 ± 21.15	0.001
Diastolic blood pressure (mmHg)	76.17 ± 10.61	79.40 ± 11.01	80.70 ± 10.71	<0.001
Waist circumference (cm)	79.24 ± 9.27	85.89 ± 9.16	88.27 ± 9.42	<0.001
**Laboratory values**				
Fasting glucose (mmo/L)	4.84 ± 0.92	5.09 ± 1.27	5.35 ± 1.53	<0.001
Serum triglyceride (mmol/L)	1.08 (0.79–1.55)	1.37 (0.96–2.05)	1.43 (1.02–2.22)	<0.001
Serum low density lipoprotein (mmol/L)	3.03 ± 0.86	3.31 ± 0.93	3.34 ± 0.93	0.048
Serum high density lipoprotein (mmol/L)	1.58 ± 0.33	1.51 ± 0.32	1.45 ± 0.32	0.47
HOMA-index (uU/mL)	1.57 (1.07–2.29)	1.98 (1.34–3.17)	2.39 (1.48–3.85)	<0.001
Urine albumin-to-creatinine ratio (mg/g)	7.96 (5.48–12.20)	8.75 (6.01–16.18)	10.25 (6.72–20.33)	<0.001
Serum uric acid (umol/L)	333.22 ± 88.99	361.33 ± 92.22	382.10 ± 105.59	0.001
Serum Creatitine (umol/L)	72.38 ± 15.78	73.41 ± 15.64	74.69 ± 19.33	0.07

Mean ± SD or median (25th to 75th percentiles) for continuous variables and proportion (95% confidence interval) for category variables are presented.

Of the subjects, 50.43% (925) had a low level of hs-CRP, 30.21% (554) subjects had an intermediate hs-CRP level and 19.36% (355) subjects had an elevated hs-CRP level. Eight subjects were receiving statin therapy and no subject was taking glucocorticoid. No subjects had a history of autoimmune disease. Subjects with a higher level of hs-CRP were older. They also had significantly higher levels of serum triglyceride, serum LDL, HOMA-index, ACR, serum uric acid, serum fasting glucose, higher systolic blood pressure and larger waist circumferences. They had higher proportions of history of hypertension and diabetes. These differences were significant (*p* < 0.05). Compared with the subjects with a low level of hs-CRP, subjects with an intermediate level of hs-CRP had significantly higher levels of serum triglyceride, serum LDL, HOMA-index, ACR, serum uric acid, serum fasting glucose, higher systolic blood pressure and larger waist circumferences (*p* < 0.05, not shown in the table). The differences in HDL and serum creatitine among three subgroups were not significant. 

### 3.2. Prevalence of Metabolic Disorders according to C-reactive Protein Categories (Table 2)

Subjects with a higher level of CRP had significant higher prevalence of metabolic disorders including components of MetS, IR, hyperuricemia and ACR. 

**Table 2:** Prevalence of metabolic disorders according to C-reactive protein categories.

Metabolic disorder % (95% CI)	Group 1	Group 2	Group 3	*p* Value
	CRP < 1 mg/L	1 mg/L < CRP < 3 mg/L	CRP > 3 mg/L	
	N = 925	N = 554	N = 355	
**Metabolic syndrome**	14.81 (12.52–17.10)	33.75 (29.80–37.70)	41.97 (36.81–47.13)	<0.001
**Insulin resistance**	15.78 (13.43–18.14)	33.21 (29.28–37.15)	44.51 (39.31–49.70)	<0.001
**Hypertension**	32.11 (29.09–35.20)	48.01 (43.84–52.19)	56.90 (51.73–62.08)	<0.001
**Diabetes**	5.84 (4.32–7.35)	9.21 (6.79–11.66)	12.96 (9.44–16.47)	<0.001
**An elevated blood pressure**	53.19 (49.97–56.41)	68.05 (64.16–71.94)	75.49 (71.00–79.99)	<0.001
**ACR**	7.35 (5.67–9.04)	13.18 (10.35–16.00)	18.31 (14.27–22.35)	<0.001
**Low HDL levels**	9.08 (7.23–10.94)	11.19 (8.59–13.82)	17.18 (13.24–21.13)	<0.001
**An elevated fasting glucose level**	9.84 (7.92–11.76)	18.72 (15.51–22.03)	23.81 (19.37–28.15)	<0.001
**Hyperuricemia**	24.43 (21.66–27.21)	36.23 (32.21–40.25)	45.94 (40.74–51.13)	<0.001
**Abdominal obesity**	32.54 (29.52–35.57)	61.37 (57.30–65.43)	70.15 (65.36–74.92)	<0.001
**Elevated triglyceride levels**	20.22 (17.62–22.81)	34.16 (30.16–38.07)	39.44 (34.33–44.55)	<0.001

### 3.3. Correlation of Log-CRP Levels with Clinical Characteristics and Metabolic Disorders (Table 3)

The distribution of CRP levels in this population was skewed to the right. Univariate regression models showed that age, history of coronary heart disease, smoking, alcohol use, education attainment, ACR and uric acid were correlated with the level of log-CRP. All of the components of MetS were positively correlated with log-CRP levels in univariate regression analysis. In the adjusted regression model, age and physical inactivity were still correlated with the level of log-CRP. Among the five components of MetS, abdominal obesity, low LDL levels, and an elevated fasting glucose level were correlated with the level of log-CRP. ACR and uric acid were also correlated with the level of log-CRP.

**Table 3:** Correlation of log-CRP levels with clinical characteristics and metabolic disorders.

Variable	Unadjusted		Adjusted	
	Linear Regression Coefficient (SE) (95% CI)	*p* Value	Linear Regression Coefficient (SE) (95% CI)	*p* Value
**Age**	0.02	<0.001	0.008	<0.001
	(0.02–0.02)		(0.004–0.012)	
**Male**	0.09	0.10	0.004	0.96
	(−0.02–0.20)		(−0.13–0.14)	
**History of coronary heart disease**	0.40	0.03	0.13	0.41
	(0.04–0.75)		(−0.19–0.46)	
**History of stroke**	0.31	0.48	−0.09	0.81
	(−0.54–1.16)		(−0.86–0.67)	
**Physical inactivity**	0.01	0.86	0.14	0.008
	(−0.10–0.12)		(0.04–0.24)	
**Education attainment**	−0.34	<0.001	−0.09	0.098
**(High school or above)**	(−0.44–−0.23)		(−0.20–0.02)	
**Current smoker**	0.20	0.02	0.16	0.059
	(0.04–0.36)		(−0.006–0.33)	
**Current alcohol use**	0.34	0.003	0.16	0.15
	(0.12–0.56)		(−0.06–0.37)	
**Elevated blood pressure**	0.49	<0.001	0.08	0.18
	(0.38–0.60)		(−0.03–0.19)	
**A low HDL level**	0.40	<0.001	0.26	0.001
	(0.24–0.57)		(0.10–0.41)	
**Abdominal obesity**	0.80	<0.001	0.57	<0.001
	(0.70–0.90)		(0.46–0.68)	
**An elevated level of blood glucose**	0.55	<0.001	0.19	0.007
	(0.40–0.69)		(−0.15–−0.34)	
**Uric acid**	0.003	<0.001	0.002	<0.001
	(0.002–0.003)		(0.001–0.002)	
**ACR**	0.24	<0.001	0.11	0.001
	(0.18–0.30)		(0.04–0.17)	

### 3.4. Association of Elevated CRP Levels with the Number of Metabolic Syndrome Components (Table 4 and Table 5)

In the total population, subjects with a higher level of CRP had a greater number of MetS components (Table 4). In the unadjusted model, compared with participants without any components of MetS, relative risks of elevated CRP levels were 2.79 (95% CI 1.71–4.59, *p* < 0.001), 4.85 (95% CI 3.00–7.84, *p* < 0.001), 6.48 (95% CI 3.97–10.58, *p* < 0.001) and 10.62 (95% CI 6.10–18.50, *p* < 0.001) for subjects with 1, 2, 3, or more than 3 components, respectively. The number of components of MetS was a significant determinant of elevated CRP levels after adjusted for age, sex, current smoking, current alcohol use, education attainment, history of coronary heart disease, history of stroke, and physical inactivity. Further adjustment for ACR and uric acid, the number of components of MetS was still significantly associated with elevated CRP levels. In multivariate analysis, relative risks of elevated CRP levels were 2.40 (95% CI 1.44–3.99, *p* = 0.001), 3.63 (95% CI 2.20–5.98, *p* < 0.001), 4.23 (95% CI 2.51–7.11, *p* < 0.001) and 6.23 (95% CI 3.45–11.26, *p* < 0.001) for subjects with 1, 2, 3, or more than 3 components, respectively.

**Table 4:** Number of metabolic syndrome components present in subjects with different CRP categories.

Number of MetS components	Group 1	Group 2	Group 3
	CRP < 0.5 mg/L	1 mg/L < CRP < 3 mg/L	CRP > 3 mg/L
	N = 925	N = 554	N = 355
**0 component**	281 (30.38)	74 (13.36)	22 (6.20)
**1 component**	301 (32.54)	132 (23.83)	75 (21.13)
**2 components**	206 (22.27)	161 (29.06)	109 (30.70)
**3 components**	110 (11.89)	135 (24.37)	97 (27.32)
**4 components**	23 ( 2.49)	47 (8.48)	41 (11.55)
**5 components**	4 (0.43)	5 (0.90)	11 (3.10)

**Table 5:** Association of an elevated CRP level with the number of metabolic syndrome components.

Number of Component	Model one ^a^		Model two ^b^		Model three ^c^		Model four ^d^	
	RR (95% CI)	*p* value	RR (95% CI)	*p* value	RR (95% CI)	*p* value	RR (95% CI)	*p* value
0 component	Reference		Reference		Reference		Reference	
1 component	2.79 (1.70, 4.59)	<0.001	2.48 (1.50, 4.11)	<0.001	2.40 (1.44, 3.98)	0.001	2.40 (1.44, 3.99)	0.001
2 component	4.85 (3.00, 7.84)	<0.001	4.19 (2.56, 6.85)	<0.001	4.02 (2.45, 6.60)	<0.001	3.63(2.20, 5.98)	<0.001
3 component	6.48 (3.97, 10.58)	<0.001	5.46 (3.30, 9.06)	<0.001	5.35 (3.22, 8.89)	<0.001	4.23 (2.51, 7.37)	<0.001
4–5 component	10.62 (6.10, 18.50)	<0.001	8.97 (5.07, 15.89)	<0.001	8.62 (4.85, 15.31)	<0.001	6.23 (3.45, 11.26)	<0.001

^a^ Unadjusted; ^b^ Adjusted for age, sex; ^c^ Adjusted for age, sex, history of coronary heart disease, history of stroke, current smoker, current alcohol use, physical inactivity, and education attainment; ^d^ Adjusted for age, sex, history of coronary heart disease, history of stroke, current smoker, current alcohol use, physical inactivity, education attainment, uric acid and ACR.

### 3.5. AUC and Optimal Cut Points of hs-CRP for MetS

The AUC of hs-CRP for MetS was 0.6954 (95% CI, 0.67–0.72). The AUC of hs-CRP for MetS was 0.67 (95% CI, 0.63–0.71) in men and 0.71 (95% CI, 0.68–0.74) in women. The optimal cut-off of hs-CRP was 0.92 mg/L (74.71%–54.06%) in men and 0.86 mg/L (77.26%–53.86%) in women.

## 4. Discussion

In this Chinese population, 50.43% subjects have a low level of hs-CRP, 30.21% (554) subjects had an intermediate hs-CRP level and 19.36% (355) subjects had an elevated hs-CRP level. Among the five components of MetS, abdominal obesity, a low level of LDL, and an elevated fasting glucose level are correlated with the level of log-CRP. Age, physical inactivity, ACR and uric acid are also correlated with log-CRP levels. The number of components of MetS is a significant determinant of elevated CRP levels after adjusted for other potential confounders including ACR and uric acid. 

Prospective epidemiologic studies indicated that hs-CRP independently predicts the mortality of CVD and CV events [29,30,31,32,33]. The largest of cohorts study is based on 27,939 initially healthy American women [31]. Hypersensitivity CRP not only adds prognostic information to the Framingham Risk Score but also links to MetS [4,34,35] and the incident of type 2 diabetes [33,35]. C-reactive protein plays an important role in the pathogenesis of atherosclerosis [36]. There are several potential mechanics [12]: (1) CRP can bind the oxidized LDL [37], (2) CRP can decrease nitric-oxide production and inhibit angiogenesis [38,39,40], (3) Synergy between CRP and inflammatory mediators might play a role in the pathogenesis of atherosclerosis [41]. (4) CRP also can activate complement [42]. 

Because of the evidence, CDC has endorsed measuring CRP in assessing the risk of CVD [14,15]. As we know, there is very limited data on CRP levels in Chinese population. The results based on a population-based cross-sectional survey conducted in Beijing during 2002–2004 indicated that the median and geometric mean concentrations of hs-CRP were 1.00 mg/L and 0.79 mg/L, respectively. A level of hs-CRP showed an upward trend with increased age (*p* < 0.05) [43]. However, this population could not represent a general Chinese population, because the population aged 45–74 years [43]. In the present study, all adults were included in the analyses. In this Chinese population, the median of hs-CRP is 1.01 mg/L and 19.47% subjects have an elevated level of hs-CRP. Compared with American, the prevalence of an elevated level of hs-CRP is lower [40].

The distribution of C-reactive protein (CRP) levels varies significantly between the various ethnic groups. According to the previous studies, we collected data of distribution of CRP in different ethnic groups and listed them in Table 6 [3,42,44,45]. Obesity is associated with the level of CRP. Both BMI and waist circumference are associated with the level of CRP [16]. The difference in BMI between the various race groups might be a potential explanation for the difference in CRP. However, this did not entirely explain CRP differences [45]. Racial difference in CRP is still controversial. In the present study, the median of CRP in the Chinese population is 0.99 mg /L. This is lower than the median of CRP in 357 Asians in the previous study [44], but is higher than that of Janpanese population [3]. Sex-difference in hs-CRP has also been reported [3], but in the present study, the difference in the male and female is not significant. This is similar to the result in the previous study based on a Chinese population [43].

**Table 6:** Distribution of CRP in different ethnic groups.

Reference	Median of CRP	Ethnic Group	Number of Subjects
Albert MA, *et al* [44]	2.96	African women	475
	2.02	Caucasian women	24,455
	2.06	Hispanic women	254
	1.12	Asian women	357
Forouhi NG, *et al* [45]	1.35	Europe	57
	0.70	South Asians	56
Sun JY, *et al* [42]	1.00	Chinese	1544
Oda E, *et al* [3]	0.28	Japanese men	1062
	0.20	Japanese women	647
Present study	0.99	Chinese	1834

Previous studies also indicated that hs-CRP is linked to MetS [4,33,34] and the incident of Type 2 diabetes [33,34]. However, the associations of hs-CRP of different components of MetS are not consistent. Not all of the components of MetS are correlated with the level of CRP [6,7,40,46,47,48]. Waist circumference is the main determinant of an elevated level of CRP [7,48]. The level of hs-CRP is increasing with the greater number of components of MetS [2,6]. In the present analysis, the results also support that not all of components of MetS are correlated with hs-CRP. Only abdominal obesity, low LDL levels, and an elevated fasting glucose level are correlated with log-CRP levels. The results also indicated that the number of components of MetS is a significant determinant of elevated CRP levels after adjusted for other potential confounders including ACR and uric acid. Urinary albumin to creatinine ratio [49] and uric acid [50] might be associated with an elevated level of CRP. The present study also supports the review. In the present study, the associations of ACR and uric acid with the level of CRP are independent of other potential confounders including components of MetS. 

Prior studies based on Japanese populations indicated that hs-CRP can be used as indicator for MetS. The optimal cutoff is 0.45 mg/L for men and 0.25 mg/L for women [3]. In the present analysis, the AUC of hs-CRP for MetS was 0.6954 for men and 0.71 for women. The optimal cut-off of hs-CRP is higher in the Chinese population. 

Several limitations of this study should be mentioned. (1) The present analysis was based on the single measurement of level of CRP without repeating tests. This is a major limitation of the present analysis. (2) This is only a cross-sectional study, and the association of CRP with metabolic disorder need to be further explored in the longitudinal study. (3) In this study, we do not have enough data on CVD (only self-reported history), so we did not assess the associations of the level of CRP with CVD. (4) This population is a median population in southern China and is not a national survey, so this population might not represent all Chinese populations. (5) In the study, the ratio of male to female is about 1:2. This sample is bias [16,17].

## 5. Conclusions

In this Chinese population, 50.43% had a low hs-CRP level, 30.21% subjects had an intermediate hs-CRP level and 19.36% subjects had an elevated hs-CRP level. Among the five components of MetS, abdominal obesity, low LDL levels, and an elevated fasting glucose level are correlated with log-CRP levels. Uric acid and ACR are also correlated with log-CRP levels. The associations of uric acid and ACR with the level of CRP are independent of other potential confounders including components of MetS. The number of components of MetS is a significant determinant of elevated CRP levels after adjusted for other potential confounders including ACR and uric acid.
